# The Willingness of Healthcare Workers in Makkah, Saudi Arabia, to Be Vaccinated Against Monkeypox and Their Knowledge About Monkeypox: A Cross-Sectional Study

**DOI:** 10.3390/vaccines14070603

**Published:** 2026-07-08

**Authors:** Khulud A. Alhazmi, Karem Ibrahem, Ahmad M. Alharbi, Abdulaziz Alsaedi, Mohammed Mufrrih, Hatoon A. Niyazi, Mohammad Y. Alqahtani, Abdullateef A. Alshehri, Eman A. Abu-Seer, Mashael S. Alfaifi, Mohammed A. Almatrafi, Rakan Ekram, Tassnym H. Sinky, Sana M. Hawsawi, Khadejah A. Ambark, Sultanah Albalawi, Dana H. Aljabri, Jana A. Noorwali, Muhannad Alsharif, Sultanah M. Alabboud, Basem A. Jawa

**Affiliations:** 1 Department of Microbiology and Parasitology, Faculty of Medicine, Umm Al-Qura University, Makkah 24382, Saudi Arabia; 2 Department of Clinical Microbiology and Immunology, Faculty of Medicine, King Abdulaziz University, Jeddah 21589, Saudi Arabia; 3 Department of Clinical Microbiology Laboratory, King Abdulaziz University Hospital, Jeddah 21589, Saudi Arabia; 4 Department of Clinical Laboratories Sciences, College of Applied Medical Sciences, Taif University, Taif 21944, Saudi Arabia; 5 Department of Medical Laboratory Sciences, Faculty of Applied Medical Sciences, King Abdulaziz University, P.O. Box 80200, Jeddah 21589, Saudi Arabia; 6 Special Infectious Agents Unit BSL-3, King Fahd Medical Research Center, King Abdulaziz University, P.O. Box 80216, Jeddah 21589, Saudi Arabia; 7 Department of Clinical Laboratory Sciences, Faculty of Applied Medical Sciences, Najran University, P.O. Box 1988, Najran 61441, Saudi Arabia; 8 Department of Epidemiology and Medical Statistics, College of Public Health and Health Informatics, Umm Al-Qura University, Makkah 24381, Saudi Arabia; 9 Department of Pediatrics, College of Medicine, Umm Al-Qura University, Makkah 24381, Saudi Arabia; 10 Department of Health Administration and Hospitals, Faculty of Public Health and Health Informatics, Umm Al-Qura University, Makkah 24381, Saudi Arabia; 11 Department of Health Promotion and Health Education, College of Public Health and Health Informatics, Umm Al-Qura University, Makkah 24381, Saudi Arabia; 12 Regional Laboratory, Makkah 25321, Saudi Arabia; 13 Division of Evolution, Infection and Genomic, School of Biological Sciences, Faculty of Biology, Medicine and Health, The University of Manchester, Manchester M11 3PL, UK; 14 College of Medicine, Umm Al-Qura University, Makkah 24382, Saudi Arabia; 15 Laboratory and Blood Bank, Alnoor Specialist Hospital, Makkah Health Cluster, Makkah 24241, Saudi Arabia

**Keywords:** healthcare workers, knowledge, monkeypox, vaccine, Makkah, Saudi Arabia

## Abstract

**Background**: Monkeypox (MP) is an emerging disease with the capacity for worldwide dissemination, endangering all nations. Healthcare workers (HCWs) frequently act as the initial point of contact for MP patients; therefore, evaluating their understanding of the disease and their perspectives on MP immunisation is crucial for effective prevention and control. This study aimed to assess HCWs’ knowledge of and attitudes toward MP and associated vaccines. **Methods:** A cross-sectional study was performed, focusing on HCWs in Makkah, Saudi Arabia. Data were collected through a standardised, self-administered online questionnaire provided in both English and Arabic. The questionnaire gathered the participants’ sociodemographic information and assessed their knowledge, opinions, and attitudes concerning MP infection and vaccination. The questionnaire included multiple-choice items with response possibilities such as “Yes”, “No” and “Don’t know”. **Results**: In total, 428 HCWs participated in this study; 60.7% had adequate knowledge of MP, while 39.3% had inadequate knowledge. Some knowledge of specific aspects of the disease was reported to be variable, with 67.5% correctly identifying MP as a viral infection, 55.1% aware of the endemic nature of the disease in parts of Western and Central Africa, and 65.0% recognising skin rash as a common symptom. In terms of attitudes towards MP vaccination, 47.9% had a positive attitude, while 52.1% had a negative attitude. In total, 65.7% of the respondents indicated that vaccination is essential to controlling MP, while only 23.1% (95% CI: 19.1–27.1%) had received vaccination. Furthermore, 57.0% reported concerns about potential adverse effects. Educational level was significantly associated with knowledge of MP (*p* = 0.001), while job title (*p* = 0.008) and years of work experience (*p* = 0.018) were significantly associated with attitudes towards MP vaccination. Age group was significantly associated with MP vaccination status (*p* = 0.002), while gender, educational level, job title, and years of work experience were not significantly associated with vaccination status. **Conclusions:** HCWs demonstrated an adequate level of knowledge about MP; however, important knowledge gaps and negative attitudes towards MP vaccination remained. Despite recognising the importance of vaccination, vaccine uptake was low. Educational level was associated with knowledge about MP, while job title and years of work experience were associated with attitudes towards MP vaccination, and age group was associated with MP vaccination status. Targeted educational interventions and evidence-based awareness programmes may enhance knowledge, improve preparedness for future MP outbreaks, and decrease vaccine hesitancy among HCWs.

## 1. Introduction

MP disease results from infection with the MP virus, which is classified as a zoonotic pathogen capable of transmitting from animals to humans [1,2]. The virus is a member of the *Poxviridae* family, specifically within the *Chordopoxvirinae* subfamily and the *Orthopoxvirus* genus. It is characterised as a double-stranded DNA virus [3,4]. Small rodents serve as the natural reservoir for MP infection, and both humans and monkeys act as hosts. The disease-causing orthopoxvirus was first identified in monkeys in 1958, and by 1970, it had been demonstrated that the virus could also infect humans [5].

Human MP illness was first identified in 1970 in a young child from the Democratic Republic of Congo. Since then, the disease has primarily been limited to West Africa and the Congo Basin. However, outbreaks have recently occurred in the United States (US) and Sudan [3], raising concerns about the potential for further spread of the disease beyond its traditional regions. MP cases have been recorded in several non-endemic countries since early May 2022. On 14 July 2022, the Saudi Ministry of Health reported the first case of MP in Riyadh, in a patient who had a history of international travel [6].

MP can spread to humans by direct contact with sick people or animals through biting, scratching, or hunting. The virus may also spread through direct contact, such as kissing, skin-to-skin contact, and face-to-face exposure involving respiratory droplets from breathing or talking. In healthcare settings, MP can be transferred through needlestick injuries and contact with infected equipment or materials. Additionally, social settings like tattoo parlours may be sites of MP transmission, particularly due to the use of shared equipment and close physical proximity between clients and artists. Vertical transmission of the virus during pregnancy and childbirth is also possible [7].

The incubation period for MP can last up to 21 days [8]. Individuals infected with MP may exhibit symptoms including a runny nose, rash, fever, stiff neck, headache, conjunctivitis, nausea, wheezing, lymphadenopathy, sore throat, chest pain, shortness of breath, sweating, back pain, vomiting, abdominal pain, muscle aches, confusion, joint pain, and coughing [9]. For most patients, these symptoms typically persist for two to four weeks and are self-limiting [10]. However, approximately 40% develop complications requiring medical intervention such as antibacterial and antiviral treatments, as well as pain management. Complications may include skin and anorectal abscesses, painful swallowing, odynophagia, penile swelling, and rectal pain [10].

There is currently no specific FDA-approved medical treatment for MP infection [11]. Recently, however, Brincindofovir, Tecovirimat, and Vaccinia immunisation globulin have been used to help control the spread of the disease [12]. Previous vaccines against smallpox have been shown to prevent MP with an efficiency rate of 85%; however, the smallpox vaccine has been unavailable since smallpox was eradicated worldwide [13]. It is possible to reduce or prevent the severity of the disease with the help of the vaccine. The MVA-BN (JYNNEOS) vaccine and the drug Tecovirimat were approved for the prevention and treatment of MP in 2019 and 2022, respectively, but their availability remains limited [14,15]. The United States has two vaccines available to reduce the severity and risk of MP disease: ACAM2000 and JYNNEOS [16]. The Centers for Disease Control and Prevention and the Advisory Committee on Immunization Practices have approved and recommend the JYNNEOS vaccine for preventing both MP and smallpox. The ACAM2000 vaccine has been approved for smallpox vaccination, which may also be used for MP immunisation [17]. The World Health Organization (WHO) recommends MVA-BN or LC16 vaccination for those at risk of MP infection during an outbreak. If other vaccines are not available for certain individuals, ACAM2000 can be used instead [18].

Reports indicate that Europe, the United Kingdom, Australia, and North America bear particularly high burdens of MP outbreaks. However, given the growing body of evidence indicating that both the severity and frequency of these outbreaks have recently risen substantially, all countries must enhance their preparedness and response capacities to effectively and comprehensively manage potential future occurrences [19]. HCWs play an active role in managing infectious diseases such as MP, particularly regarding diagnosing, treating, and preventing the spread of the virus. HCWs also play a crucial role in educating the public and promoting disease prevention measures. Encouraging HCWs to take the vaccine and educating them about the MP-associated risk factors, early symptoms, and protective measures is critical to the early recognition and containment of the disease. HCWs exhibiting a lack of knowledge of MP and sceptical attitudes towards vaccines can hinder effective patient care and public health efforts. A deeper understanding of these gaps is crucial to improving preparedness and response strategies, since early detection can lead to improved patient outcomes and reduced transmission. Outbreaks of MP in non-endemic countries highlight the importance of preparedness and rapid response to the prevention of widespread transmission and future outbreaks. The strategic significance of this study is highlighted by the special status of the city of Makkah as a destination for millions of visitors from different geographical backgrounds, which may include regions endemic for MP. As HCWs in Makkah are the first responders to cross-border infectious diseases, their vigilance and readiness to respond are crucial. HCWs with an elevated awareness of MP and a vaccination-friendly attitude are not only requirements for the city of Makkah but are also important for global health. Awareness and openness to vaccination among this workforce are essential to avoiding possible outbreaks during mass gathering seasons, when the risk of the virus being introduced and spreading is high.

Consequently, this study aimed to assess healthcare workers’ knowledge, awareness, and attitudes regarding MP and associated vaccines.

## 2. Methods

### 2.1. Subjects, Inclusion and Exclusion Criteria

A cross-sectional study was conducted among healthcare workers (HCWs) in the Makkah region of Saudi Arabia using an electronically distributed questionnaire. A convenience sampling method was used by distributing an online English-language questionnaire via email and social media platforms, including Instagram, WhatsApp, Facebook, and Twitter, to HCWs in the Makkah region of Saudi Arabia.

All healthcare workers, including males and females as well as Saudi and non-Saudi nationals, were eligible to participate. Individuals who were not healthcare workers or who were working outside the Makkah region were excluded from the study.

For the purposes of this study, HCWs included licensed healthcare professionals as well as healthcare students, interns, trainees, and volunteers who were involved in healthcare-related education or clinical settings within the Makkah region.

### 2.2. Study Variables

The questionnaire used in this study is divided into three main sections: the first section includes the sociodemographic characteristics of the participants (such as gender, age, marital status, nationality, education level, job title, and work experience); the second section focuses on assessing HCWs’ knowledge of MP; and the last section is designed to evaluate participants’ attitudes towards MP and associated vaccines. The questionnaire consists of multiple-choice questions with the following response options: “Yes”, “No”, and “I do not know.”

For content validity, two experts in virology reviewed the questionnaire. A pilot study was conducted with 30 HCWs to determine the clarity of the questions and the time needed to complete the questionnaire. The feedback received was used to make some minor changes for clarity. The information obtained in the pilot study was not included in the final analysis.

### 2.3. Sample Size

The sample size was calculated using the Raosoft calculator, using all HCWs in Makkah as the population size (http://www.raosoft.com/samplesize.html, accessed on 30 March 2025). The required number of samples, with a 5% margin of error and a 95% confidence interval, was 385. The final sample size was adjusted by approximately 10% to account for non-responders and incomplete submissions.

### 2.4. Data Analysis

Participant responses were entered into Microsoft Excel and then imported into IBM SPSS Statistics version 30.0 (IBM Corp., Armonk, NY, USA) for statistical analysis. The data were summarised using descriptive statistics. Frequencies and percentages were calculated for the sociodemographic characteristics, knowledge, attitudes, and vaccination status of the participants. No missing data were identified among the completed questionnaires included in the final analysis.

Knowledge Score Calculation: One point was awarded for each correct response to the knowledge-oriented questions. The total knowledge score for each participant was calculated by summing the correct responses across the 10 knowledge items, resulting in possible scores ranging from 0 to 10. Participants who scored 5 or more were considered to have adequate knowledge, whereas those scoring below 5 were classified as having inadequate knowledge. The cut-off value was determined based on the median knowledge score observed in the study population.

Attitude Score Calculation: A positive response for each attitude item was based on the intended direction of the questions. Responses indicating support for MP vaccination were assigned a score of 1, whereas all other responses were scored as 0. The scores for the nine attitude items were summed to obtain the total attitude score, yielding possible scores ranging from 0 to 9. Participants who scored 5 or above were classified as having a positive attitude towards MP vaccination, while those scoring below 5 were considered to have a negative attitude. The cut-off value was based on the median attitude score observed in the study population.

To examine the associations between participants’ knowledge level, attitude, and vaccination status and their sociodemographic characteristics (age, sex, education level, job title, and years of experience), inferential statistical analyses were performed.

Associations between knowledge level (adequate vs. inadequate), attitude (positive vs. negative), and categorical sociodemographic variables were assessed using Pearson’s chi-square test. Vaccination status was analysed using the three original response categories (“Yes”, “No”, and “I do not know”), and its association with the sociodemographic variables was also examined using Pearson’s chi-square test. A two-sided *p*-value < 0.05 was considered statistically significant. IBM SPSS Statistics version 30.0 (IBM Corp., Armonk, NY, USA) was used for all analyses. 

### 2.5. Ethical Approval

The participants were HCWs at different healthcare providers in Makkah, Saudi Arabia. They were required to provide informed consent by clicking on the consent statement before completing the questionnaire. The consent statement stated the aims and purpose of the investigation. HCWs who agreed to participate in the study completed the questionnaires. The study received approval from the Biomedical Research Ethics Committee of Umm Al-Qura University in Makkah, Saudi Arabia (Approval No. HAPO-02-K-012-2024-11-2336).

## 3. Results

### 3.1. Demographic Characteristics

This study involved 428 individuals. Table 1 presents a comprehensive demographic profile of these individuals. In total, 77.6% (332) of the participants were female, representing a significant majority; the remaining participants were male. The age distribution indicated that 66.8% (286) of the cohort were aged between 20 and 25. After examining marital status, it was reported that a significant majority, 73.8% (316 participants), identified as single, whilst 26.2% (112) were married. Of the participants, 88.8% (380) were Saudi nationals, while 11.2% (48) were non-Saudi. Regarding educational qualifications, 55.1% (236) had a bachelor’s degree, whereas 8.4% (36) had a PhD or an equivalent qualification. The participants’ work experience in hospital settings varied; 30.6% (131) reported five or more years of experience, while 69.4% (297) reported less than five years. Regarding their professional roles in the hospital setting, the participants were categorised as follows: 29.9% (128 individuals) were physicians, 12.9% (55 individuals) worked in laboratory settings, and 11.7% (50 individuals) were nurses. The remaining 45.6% (195 individuals) were internship students from different disciplines, students from different medical fields, trainees, and volunteers in hospitals and health centres (Table 1).

### 3.2. Monkeypox Knowledge and Awareness

Table 2 presents an overview of the participants’ general knowledge concerning MP. Approximately half of the respondents (52.6%, *n* = 225) accurately recognised that MP is not caused by a bacterial agent, and more than half (67.5%, *n* = 289) correctly identified MP as a viral infection. Less than half of the participants (33.9%, *n* = 145) were aware that MP is not prevalent across Middle Eastern nations, while 25.5% (*n* = 109) responded that it is, and the rest of the participants did not know. About half (55.1%, *n* = 236) believed that the disease is endemic to certain parts of Western and Central African regions. Additionally, only a small percentage (36%, *n* = 154) stated that they were aware of the limited number of cases of human MP previously reported in Saudi Arabia.

Regarding the clinical features of the disease, slightly more than half of the participants (51.9, *n* = 222) believed that the symptoms and signs of MP are similar to those of smallpox. Furthermore, a significant percentage (65%, *n* = 278) were aware that skin rashes are one of the common signs of MP infection, and 50.7% (*n* = 217) believed a flu-like syndrome is one of the signs of the infection. Regarding transmission, more than half of the participants (56.5%, *n* = 242) were aware that MP is easily spread from one person to another; however, only 40.4% (*n* = 173) correctly acknowledged that MP infections do not require antibiotic treatment.

The aggregated knowledge scores were used to classify the participants as having either adequate or inadequate knowledge of the disease, with a cut-off score of 5 points. In total, 60.7% (260) of the participants had adequate knowledge of MP, whereas 168 (39.3%) were considered to have inadequate knowledge (Table 3).

### 3.3. Perceptions of and Attitudes Towards Monkeypox Vaccines

Table 4 summarises the participants’ opinions, awareness, and attitudes regarding the MP vaccine. Most respondents (65.7%, *n* = 281) expressed the belief that vaccination is necessary for controlling the spread of MP infection. Approximately 53.3% (*n* = 228) of the participants indicated concerns regarding potential adverse effects of the MP vaccination, while nearly 48.4% (*n* = 207) expressed concerns about its efficacy.

Significantly, just 50.7% of the respondents (*n* = 217) expressed a willingness to encourage their friends and families to be vaccinated against MP. Also, less than half (43.5%, *n* = 186) said they are confident in the overall safety of vaccines. Despite these concerns, over half (54.9%, *n* = 235) agreed that the advantages of vaccination exceed the related risks, and 48.4% (*n* = 207) expressed trust in the vaccine’s efficacy in protecting against MP. When questioned about their vaccination intentions, just over half (52.3%, *n* = 224) expressed a willingness to receive an MP vaccine, and only 23.1% (*n* = 99) had been vaccinated at the time of the survey. Additionally, 44.9% (*n* = 192) believed that MP could trigger a global pandemic.

Participants were categorised as either having a positive or negative attitude based on the sum of the attitude scores, with the cut-off score assigned to be 5. In general, the attitudes of the 205 participants were positive towards MP vaccination (47.9%), and 223 participants were considered as having negative attitudes (52.1%) (Table 5).

### 3.4. Monkeypox Vaccination Perspectives

**Figure 1:**
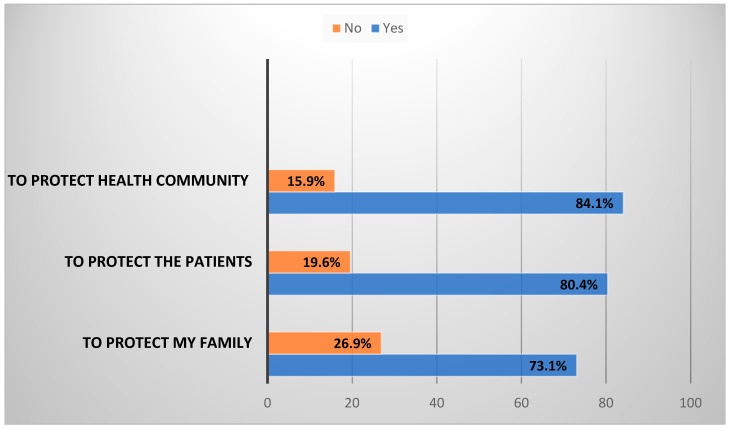
Participants’ reasons for agreeing to receive the monkeypox vaccine.

Figure 1 illustrates the participants’ explanations of their reasons for agreeing to receive the MP vaccine. Most respondents, 84.1% (*n* = 360), indicated that their primary motivation for vaccination was to contribute to the protection of community health. This was followed by 80.4% (*n* = 344) agreeing that the safety of patients was a significant factor influencing their willingness to be vaccinated. Additionally, 73.1% (*n* = 313) cited the protection of their families as a key reason for receiving the MP vaccine.

### 3.5. Monkeypox Vaccination Hesitancy

**Figure 2:**
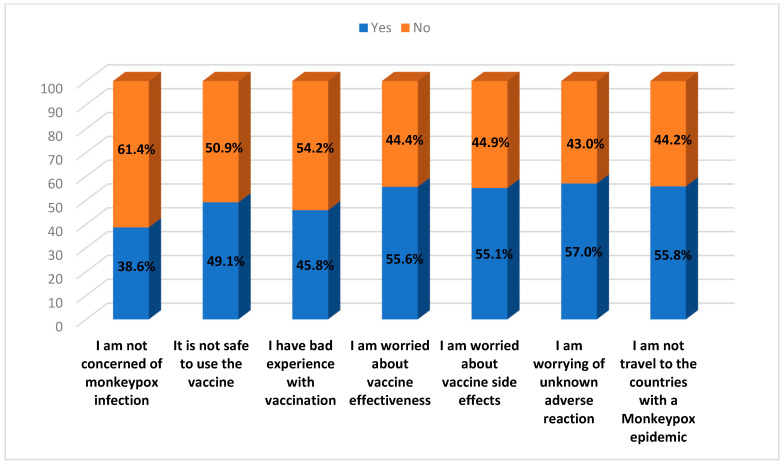
Participants’ rationale for declining to receive the monkeypox vaccine.

Figure 2 presents the participants’ reported reasons for hesitancy or refusal to receive the MP vaccine. More than half of the respondents, 57% (*n* = 244), expressed concerns about potential unknown adverse reactions associated with the vaccine. Likewise, 55.8% (*n* = 239) asserted that immunisation was unnecessary for them, as they did not visit regions where MP is endemic. Additionally, 55.6% (*n* = 238) were concerned about the vaccine’s efficacy. In contrast, 55.1% (*n* = 236) expressed concerns about the potential side effects. Nearly half of the participants, 49.1% (*n* = 210), regarded immunisation as hazardous. Moreover, 45.8% (*n* = 196) indicated that prior negative experiences with immunisation had influenced their reluctance to receive the MP vaccine. Ultimately, 38.6% (*n* = 165) did not perceive MP infection as a significant personal issue, which intensified their reluctance to receive the vaccine.

### 3.6. Monkeypox Vaccination Status

Among the 428 HCWs who participated in the study, 99 participants (23.1%; 95% CI: 19.1–27.1%) reported having received the MP vaccine. Additionally, 222 participants (51.9%) stated that they had not received the vaccine, whereas 107 participants (25.0%) indicated that they did not know whether they had received the MP vaccine.

### 3.7. Classification of Knowledge Level, Attitudes, and Vaccination Status

According to the predefined scoring criteria, 260 participants (60.7%) had adequate knowledge of MP, whereas 168 participants (39.3%) had inadequate knowledge. In addition, 205 participants (47.9%) expressed positive attitudes towards MP vaccination, whereas 223 participants (52.1%) demonstrated negative attitudes.

Regarding MP vaccination status, 99 participants (23.1%) indicated having received the MP vaccine, 222 participants (51.9%) reported that they had not received the vaccine, and 107 participants (25.0%) indicated that they did not know whether they had received the MP vaccine.

### 3.8. Association Between Knowledge Level and Sociodemographic Characteristics

Pearson’s chi-square test was used to examine the association between the knowledge level exhibited by HCWs and selected sociodemographic characteristics of the study participants. The participants were categorised as having adequate or inadequate knowledge, depending on their total knowledge score. Educational level had a significant association with knowledge level (*p* = 0.001), as shown in Table 6. However, there were no statistically significant differences in knowledge level based on gender (*p* = 0.605), age group (*p* = 0.070), job title (*p* = 0.086), or years of experience (*p* = 0.986).

### 3.9. Association Between Attitudes Towards Monkeypox Vaccination and Sociodemographic Characteristics

Pearson’s chi-square analysis was conducted to examine associations between HCWs’ attitudes towards MP vaccination and selected sociodemographic characteristics. Participants were categorised as having either a positive or a negative attitude based on their total attitude score. A statistically significant association was observed between attitude towards MP vaccination and job title (*p* = 0.008), as well as years of work experience (*p* = 0.018), as presented in Table 7. However, no statistically significant associations were found between attitude and gender (*p* = 0.299), age group (*p* = 0.172), or educational level (*p* = 0.078).

### 3.10. Association Between Monkeypox Vaccination Status and Sociodemographic Characteristics

Pearson’s chi-square test was used to investigate the associations between MP vaccination status and selected sociodemographic characteristics. Vaccination status was analysed using the three original response categories (“Yes”, “No”, and “I do not know”) as reported by the participants. As indicated in Table 8, age group was significantly associated with MP vaccination status (*p* = 0.002). However, no statistically significant associations were observed between vaccination status and gender (*p* = 0.382), educational level (*p* = 0.142), job title (*p* = 0.056), or years of work experience (*p* = 0.051).

## 4. Discussion

Several non-endemic areas, including countries in North America, Europe, and Oceania, have reported an increased number of MP cases. These unexpected routes of transmission have sparked widespread public health fears, suggesting an unusual and potentially growing threat. This is especially alarming because over 70% of the world’s population remains unvaccinated against smallpox, and this translates into decreased cross-protective immunity to MP, consequently increasing population susceptibility during epidemics [19]. The incidence of MP epidemics in non-endemic countries underscores the critical need for thorough preparedness measures to prevent the disease’s transition to an international pandemic. For the management and containment of MP to be effective, healthcare providers must have adequate knowledge of and positive attitudes toward community transmission, and must adopt appropriate practices to reduce it [20].

Although the MP outbreak has had a less substantial impact on daily life than the COVID-19 pandemic, concerns remain regarding the virus’s ability to evolve and its potential public health implications. Studies indicate that the MP virus is actively evolving in human hosts, raising concerns about its potential to generate more transmissible or clinically significant variants [21]. The insights that were gained during the COVID-19 pandemic emphasise the need to achieve a deeper understanding of all aspects of infectious diseases in general and MP sickness more specifically. In addition, they emphasise that the proactive development and implementation of strategies in anticipation of potential future outbreaks are also more successful than managing illness in infected patients once an outbreak occurs. Timely and appropriate intervention by HCWs is necessary to successfully address the current MP outbreak. To support this initiative, it is necessary to evaluate whether healthcare staff are currently aware of MP. With this assessment, awareness can be raised and specific training regarding health worker skills can be offered, which will equip them with the necessary knowledge and techniques needed to effectively manage and prevent the spread of MP. This can increase their preparedness for response to the growing threat of MP and the effective mitigation thereof, as advised by the WHO [22]. HCWs are a risk group in the current human MP outbreak because they are at risk of contracting the disease and have the potential to spread the virus to the rest of the population. Hence, it is of utmost importance to assess their knowledge of the disease and associated vaccination programmes. Identified gaps in knowledge can then be effectively addressed using appropriate educational and training initiatives. Moreover, accurate information and practical training for HCWs can enhance the quality of the care they provide, which is crucial to ensuring safe and high-quality care for patients [23].

This study was conducted in the city of Makkah, Saudi Arabia, in order to investigate HCWs’ knowledge, awareness, and attitudes concerning MP and associated vaccines. In this study, only 52.6% of the respondents correctly stated that MP is not related to a bacterial aetiology, and 67.5% were aware that it is a viral infection. A study on HCWs and medical students conducted in 2024 reported that 76% of respondents correctly identified MP as non-bacterial. Additionally, 83.7% were aware that MP is an infectious viral disease that is a zoonosis [24]. In addition, a 2023 study found that most respondents were able to correctly identify MP as a viral (78.9%) rather than bacterial (65.6%) disease [20]. In this study, 33.9% of the participants recognised that MP is not endemic to Middle Eastern countries. Furthermore, 36% showed awareness of the small number of documented cases of MP in Saudi Arabia. A 2022 survey by Alshahrani et al. revealed that many participants perceived MP to be uncommon in the Middle East, and over three-quarters were unaware of any cases in Saudi Arabia [25]. Moreover, 55.1% correctly recognised that the disease is common in specific areas of Western and Central Africa. This data aligns with a 2024 study by Amer et al., which indicated that 52.2% of participants acknowledged the disease’s prevalence in these African locations [24]. Similar findings have been reported among healthcare professionals in other countries, where awareness of the geographical distribution of MP and its occurrence outside endemic regions was variable. These findings suggest that gaps in epidemiological knowledge persist among HCWs despite their professional background [20]. Moreover, in this study, 51.9% of the participants indicated that MP presents signs and symptoms analogous to those of smallpox. Furthermore, 65% recognised that skin rashes are common symptoms of MP infection. Conversely, another study revealed that a significant majority of participants, 91.2%, were oblivious to the fact that MP exhibits analogous signs and symptoms to smallpox, although most individuals, 72.9%, recognised skin rashes as signs and symptoms linked to MP [20]. Furthermore, over half of the participants (56.5%) recognised that MP can be easily transmitted from person to person, while 73.3% identified close contact as a mode of transmission in a 2024 study [24]. Additionally, 58.8% of the participants in another study understood that MP could be transmitted between humans [20]. In the present study, less than half of the participants (40.4%) correctly stated that antibiotic treatment is ineffective against MP infections. Conversely, Sobaikhi et al. revealed that a substantial 64.8% of their cohort incorrectly favoured using antibiotics to treat the virus [20]. This highlights a potential fallacy regarding how viral diseases can be treated since antibiotics do not treat viruses and are instead usually used to treat bacterial infections.

Our study revealed that some of the participants had gaps in their knowledge regarding the virus. This aligns with findings from research conducted in Bangladesh, where only 31% of medical doctors could accurately answer 70% of the questions posed [26]. Furthermore, research conducted in Jordan revealed insufficient understanding regarding MP among healthcare professionals and medical students [27]. Comparable levels of understanding of these elements were previously noted in research conducted in Indonesia in 2020 [28].

The aggregate mean scores of the knowledge questions showed that 60.7% of the participants had an adequate level of knowledge of MP, while 39.3% were categorised as having inadequate knowledge. The total level of knowledge across HCWs was moderate, but a high proportion of knowledge deficiencies regarding the disease was evident. This is a significant discovery given the critical roles HCWs play in disease recognition, patient education, infection prevention, and outbreak response. The reported deficiencies may be due to the small number of reported cases of MP in Saudi Arabia and the relatively low exposure of HCWs to MP in their daily clinical work. Thus, targeted educational initiatives and ongoing professional development activities could be useful to boost preparedness for potential future outbreaks.

The level of knowledge and awareness among the participants in this study may be attributed to several factors, including the low and limited number of reported MP cases in Saudi Arabia, which could lead to a general deficiency of awareness of and attention to the disease outside its endemic regions [21]. Sociodemographic factors, such as years of professional experience and age, as well as healthcare profession and specialty, are also important factors that could influence the observed level of knowledge [29,30]. The study reports that 66.8% of participants aged 20–25 and 69.4% of all participants had less than five years of work experience. Previous research has indicated that individuals over the age of 40 demonstrate the highest levels of knowledge, as corroborated by statistical analysis. The younger participants, who were born after the eradication of smallpox, have experienced a diminishing emphasis on poxviruses in both training programmes and university curricula. Moreover, younger people (below 40 years old) have stated that they had less experience in related fields than their older counterparts, which is consistent with the results of previous studies [24,31,32]. The global smallpox immunisation program ended in 1980 [33], and HCWs have found themselves operating in a smallpox-free era, which is part of the reason why they do not pay much attention to the MP virus. Moreover, within normal clinical practice, many HCWs have never dealt with patients with MP, and this may have contributed to the lack of practical experience. Evidence from Indonesia suggests that local HCWs have considerably greater understanding of the most common infectious diseases, including dengue and Zika, compared to the newcomers [34,35]. Harapan et al. found that less than 20% of general practitioners had experienced MP during their medical training, which shows a gap in teaching on MP in the medical curriculum [28]. The literature shows that some healthcare professionals rely mostly on social media, as opposed to peer-reviewed scientific publications or healthcare institutions, to receive information on MP [26,36]. This reliance on social media may lead to the dissemination or reception of inaccurate information, which, in addition to weakening HCWs’ understanding of MP, can also have negative impacts on whether they are willing to receive immunisation. Knowledge improvement and creating awareness of MP among HCWs can be of great importance to enhancing compliance with infection control practices [37]. Notably, a significant proportion of participants (45.6%) held various specialised positions within the hospital, such as physical therapists, radiology technicians, dentists, interns, internship students from different disciplines, undergraduate students in various medical fields, trainees, and volunteers. The gap in awareness could be due to the relative distance between the participants’ specialities and the topic of the virus, or because some students had not yet completed their studies or gained substantial practical experience in the hospital. This may have influenced the study’s findings by lowering the average level of knowledge among the sample. In addition, Pearson’s chi-square analysis showed that there was a significant relationship between educational level and knowledge about MP. Participants who had attained a higher level of education were more likely to have adequate knowledge of the disease, which could be attributed to increased exposure to the scientific and professional literature and other evidence-based sources of information.

Regarding the attitude of the participants towards the MP vaccine, 65.7% of the respondents reported that they needed to be vaccinated to control MP infection. Nevertheless, a similar survey was carried out in 2022, and only 33.3% of the participants knew that there is a vaccine that prevents MP [27]. In this study, 50.7% of the participants reported they would support their friends and family receiving the MP vaccine, 43.5% conveyed trust in the vaccine’s safety, and 54.9% asserted that the advantages of vaccination exceed its possible dangers. Research conducted in 2024 revealed that 59.5% of participants were willing to recommend the MP vaccine to others; however, a majority (55.5%) expressed uncertainty over its safety. Moreover, 45.6% of participants in the same study agreed that the advantages of vaccination exceed the possible adverse effects [24]. In addition, 52.3% of those who participated in this study said they would agree to receiving the MP vaccine. The overall aggregated attitude scores revealed that 47.9% of the participants held positive attitudes towards MP vaccination, while 52.1% held negative views. The result indicates that there is a significant level of vaccine reluctance among HCWs. Concerns regarding vaccine safety, effectiveness, and potential adverse effects may have contributed to these attitudes. These issues are likely to impact confidence in MP vaccination and uptake, and evidence-based educational campaigns could help address these concerns. These findings show that whilst most participants exhibited some awareness of the need for vaccination, vaccine safety and efficacy remain factors in attitudes to the available vaccines. A study conducted in 2024 also showed that 56.1% of participants consented to receive the MP vaccine [24]. The inferential analysis also showed that attitudes towards MP vaccination were significantly associated with job title and years of work experience. Variations in how vaccines are perceived may be partly due to the different roles HCWs play, as well as their training, clinical experience, and knowledge of infectious diseases. In addition, a recent study conducted among Italian physicians revealed a slightly positive attitude towards MP vaccination [29]. Similarly, a study by Han et al. (2024) indicated that 54% of healthcare professionals expressed a willingness to receive the MP vaccine [38]. Likewise, a separate study involving Turkish physicians revealed that 31.4% planned to take the vaccine [39]. In the current survey, 44.9% of the participants thought that a global MP pandemic is possible. Previous studies among healthcare professionals have also reported concerns regarding the potential public health impact of MP outbreaks, although the perceived risk varies across settings and professional groups [39]. Moreover, 53.3% of the participants were concerned about the possible side effects of an MP vaccine, and 48.4% were concerned about its effectiveness. The Adult National Immunisation Survey on Immunisation showed that 18% of adults were hesitant or resistant to vaccination, with 6.5% of the non-vaccinated adults stating that they refused to receive one or more vaccinations, with the main reason given being concern about the safety of the vaccines and their side effects [40]. In comparison, another study identified that between 67.52% and 66.31% of HCWs had a positive perception towards the safety and efficacy of vaccines, respectively [41]. In the current study, the proportion of participants who had received the MP vaccine was 23.1% (95% CI: 19.1–27.1%). The vaccination coverage observed in this study was relatively low. This reduced uptake might be due to the reduced perceived threat, concerns about vaccine safety and effectiveness, or vaccine shortages during the study period. As HCWs are among the groups at increased risk of occupational exposure to infectious diseases, efforts to enhance awareness of MP and improve access to vaccination programmes are needed. Age group was significantly associated with MP vaccination status, whereas gender, educational level, job title, and years of work experience were not significantly associated. This finding may reflect differences in vaccination responses across age groups, which could be explained by variations in perceived occupational risk, awareness of MP, previous vaccination experiences, or access to vaccination opportunities. Nevertheless, vaccination decisions are likely influenced by factors beyond sociodemographic characteristics, including perceived risk of infection, confidence in vaccine safety and effectiveness, personal beliefs, and access to reliable information. These findings highlight the importance of considering behavioural and perceptual factors, in addition to demographic characteristics, when designing strategies to improve MP vaccine uptake among healthcare workers. The findings may be explained by various factors observed in previous research, such as concerns regarding vaccine safety and effectiveness, perceptions of a low risk of infection, and limited knowledge of MP. Previous studies have reported that a greater understanding of an infectious disease may be associated with higher vaccine acceptance [42,43,44]. Moreover, people often demonstrate increased anxiety about illnesses such as COVID-19 in comparison with MP [45]. In polling HCWs on their perceptions of the threat of MP, Ricco et al.’s results indicate that participants deemed this disease to be less threatening than other infectious diseases, including COVID-19, tuberculosis, acquired immune deficiency syndrome, and hepatitis B [29]. In addition, concerns regarding vaccine safety and effectiveness may contribute to reluctance by the medical fraternity. In the US, the reservations HCWs expressed pertaining to vaccination against COVID-19 included scepticism regarding its effectiveness (37.1%), safety (55.0%), and potential adverse effects in the long run (57.1%) [46]. In the article by Hong et al., 67.15% of the participants were concerned about the effectiveness of the MP vaccine, and 68.90% were concerned about its safety. Moreover, due to the self-limiting nature of MP and the fact that most cases of this illness are expected to disappear in 14–21 days [47,48], not all HCWs will be cautious and may assume that there is no need to be vaccinated even when exposed to the virus. Additionally, the mild side effects of earlier vaccinations alleviated some of the concerns that certain HCWs had regarding the safety of the MP vaccine. The history of various vaccination types may influence an individual’s behaviour, including their attitude towards vaccination in the future [49].

The respondents provided several reasons explaining why they were willing to receive the MP vaccine. A large majority (84.1%, *n* = 360) of the participants cited contributing to the safety of the population as their primary reason for agreeing to receive the vaccine. Furthermore, 80.4% (*n* = 344) reported that patient protection is an essential reason for their vaccination choice. The safety of their family was also among the most important reasons provided by 73.1% (*n* = 313) of the respondents. Conversely, the participants identified several factors that affected their reluctance to receive the MP vaccine. Additionally, 57% (*n* = 244) feared a potential undesirable impact. Similarly, 55.8% (*n* = 239) considered immunisation unnecessary since they do not travel to places where MP is endemic. Furthermore, 55.6% (*n* = 238) expressed worries regarding the efficacy of the vaccine, and 55.1% (*n* = 236) expressed worries about its side effects. Almost half (49.1, *n* = 210) considered the vaccine unsafe. Moreover, 45.8% (*n* = 196) stated that they were not willing to be vaccinated due to a negative vaccination experience in the past. Finally, 38.6% (*n* = 165) did not consider MP infection to be a major individual risk, which also contributed to their hesitation to be vaccinated.

Multiple studies have shown that HCWs are perceived by the general population to be the most trustworthy source of information about MP, and that they are involved in the promotion of vaccine acceptance. Nevertheless, when HCWs themselves are reluctant to be vaccinated, the same reluctance may be transferred to patients [29,50,51]. To address this issue, MP should be incorporated into medical programmes, and the continued education of practising HCWs should be reinforced and supported. Indeed, an awareness programme in Brazil, had increased the rate of influenza vaccination in HCWs to 34.4%, but this figure fell to 20.2% and, finally, to 12.75% when education was not continuous [52]. In addition, it is necessary to prioritise the use of scientific journals as a reliable source of information to enable HCWs to improve their of the safety and side effects of the MP vaccine and, as a result, contribute to an increase in vaccine uptake [53].

The findings of this study point to practical implications for healthcare institutions. The observed knowledge gaps and vaccine hesitancy among HCWs underscore the importance of targeted learning programmes on MP’s epidemiology, transmission, prevention, and vaccination. Ongoing training sessions, continuous education/certification courses, and evidence-based awareness campaigns could help enhance the capacity of HCWs to respond to future outbreaks and guide the education of patients and the general public.

From a public health policy perspective, therefore, the results support the incorporation of emerging infectious diseases, such as MP, into the healthcare training curricula and institutional preparedness plans. Healthcare authorities should communicate accurate and timely information about MP and associated vaccines and should make the MP vaccine available to HCWs, especially those at higher risk of occupational exposure. Enhancing surveillance, education, and vaccination policies can help to increase preparedness and improve response to outbreaks.

Interventions may be especially useful for healthcare professionals with limited education and for professional groups that have less positive attitudes regarding MP vaccination. Vaccine-specific education resources covering vaccine safety, effectiveness, and potential adverse effects may help to mitigate vaccine hesitancy and increase vaccine acceptance among HCWs.

## 5. Strengths and Limitations

This study has several strengths. First, it compared HCWs of different professions, and offered a detailed understanding of their knowledge, attitudes, and vaccination status pertaining to MP in a healthcare context. Second, this study not only explores knowledge and attitudes, but also actual vaccination uptake and the reasons for vaccine acceptance and hesitancy, thereby providing a more comprehensive perspective on the factors that influence MP vaccine decision-making. Third, a structured questionnaire the content of which was found to be valid via expert review, enhanced the quality and relevance of the collected data. Lastly, the inferential statistical analyses enabled the identification of sociodemographic factors associated with knowledge of and attitudes towards MP vaccination, as well as MP vaccination status.

The findings of this study are also subject to several limitations. To gain a better understanding of HCWs’ knowledge, attitudes, and behaviours regarding MP, future studies should use more representative sampling methods, longitudinal designs, and qualitative methods.

Sampling Method: A convenience sampling method was used through an electronically distributed survey. This could have created selection bias because only HCWs with internet access and social media accounts were likely to participate. Some sub-groups of HCWs, particularly those who are less digitally connected and belong to older age groups, might therefore have been under-represented. In addition, a large portion of the sample comprised students, interns, trainees, and volunteers. These are individuals engaged in healthcare-related learning rather than clinical practice; they may have had some effect on estimates of knowledge and attitudes as a whole and may not be representative of fully qualified healthcare professionals.

Self-Reported Data: Data collected on a self-administered questionnaire and may be affected by recall bias and social desirability bias. The participants may have over- or under-reported their knowledge, attitudes, or vaccination status.

Generalizability: The study was performed in the city of Makkah in Saudi Arabia, which has specific demography and health system characteristics. Therefore, it may not be possible to generalise the results to HCWs in other parts of Saudi Arabia.

Cross-Sectional Design: The cross-sectional design means that the changes in knowledge and attitudes over time cannot be assessed, and the relationship between variables is not causal.

Measurement Limitation: Although the questionnaire was expert-validated, it may not fully reflect the full knowledge of or subtle attitudes towards MP and associated vaccines. Variations in responses of some participants, including students and volunteers who may have limited clinical experience, may be possible.

Future studies need to include psychometric testing to further assess questionnaire reliability and validity.

## 6. Conclusions

This study aimed to assess the knowledge and attitudes of HCWs in Makkah, Saudi Arabia, regarding MP, as well as their vaccination status. Participants showed good knowledge of MP (60.7%) but a significant proportion of HCWs exhibited important knowledge gaps. Vaccine hesitancy (47.9%) was identified in less than half of the participants, due to their negative attitudes towards MP vaccination. In addition, 23.1% of the participants indicated that they had been vaccinated for MP.

Inferential analysis indicated that educational level was significantly associated with knowledge of MP, while job title and years of work experience were significantly associated with attitudes towards MP vaccination. Furthermore, age group was significantly associated with MP vaccination status, while gender, educational level, job title, and years of work experience were not significantly associated with vaccination status.

These findings highlight the importance of targeted training programmes, ongoing training courses, and evidence-based awareness campaigns to further enhance HCWs’ knowledge of and confidence in MP vaccination. Increased efforts to improve training programmes and provide evidence-based information could help mitigate knowledge gaps, address vaccine hesitancy and other factors, and improve readiness regarding new outbreaks of emerging infectious diseases.

## Figures and Tables

**Table 1:** Demographic characteristics of the participants: gender, age, marital status, nationality, education level, and occupation.

Parameters	Category	% (Number)
Gender	Male	22.4 (96)
	Female	77.6 (332)
Age	20–25	66.8 (286)
	26–45	24.3 (104)
	46–60	8.9 (38)
Marital status	Single	73.8 (316)
	Married	26.2 (112)
Nationality	Saudi	88.8 (380)
	Non-Saudi	11.2 (48)
Educational level	PhD or equivalent	8.4 (36)
	Master’s	10.3 (44)
	Bachelor’s	55.1 (236)
	Diploma	8.6 (37)
	Other	17.5 (75)
Work experience	Less than 5 years	69.4 (297)
	5 years or more	30.6 (131)
Job in hospital	Physician	29.9 (128)
	Laboratory	12.9 (55)
	Nurse	11.7 (50)
	Other	45.6 (195)

**Table 2:** Participants’ general knowledge and awareness of MP.

Parameters	Category	% (Number)
Is monkeypox a bacterial infection?	Yes	18.5 (79)
	No	52.6 (225)
	I do not know	29 (124)
Is monkeypox a viral infection?	Yes	67.5 (289)
	No	8.4 (36)
	I do not know	24.1 (103)
Is monkeypox common in the Middle Eastern countries?	Yes	25.5 (109)
	No	33.9 (145)
	I do not know	40.7 (174)
Is monkeypox common in certain parts of Western and Central Africa?	Yes	55.1 (236)
	No	11.2 (48)
	I do not know	33.6 (144)
Have any human monkeypox cases been reported in Saudi Arabia?	Yes	36 (154)
	No	23.4 (100)
	I do not know	40.7 (174)
Smallpox and monkeypox have similar signs and symptoms.	Yes	51.9 (222)
	No	12.6 (54)
	I do not know	35.5 (152)
A flu-like syndrome is one of the early signs or symptoms of monkeypox infection in humans.	Yes	50.7 (217)
	No	11.7 (50)
	I do not know	37.6 (161)
Rashes on the skin are one of the signs or symptoms of monkeypox infection in humans.	Yes	65 (278)
	No	7 (30)
	I do not know	28 (120)
Is monkeypox easily transmitted from person to person?	Yes	56.5 (242)
	No	12.4 (53)
	I do not know	31.1 (133)
Humans with monkeypox infections require antibiotics for treatment.	Yes	32.2 (138)
	No	40.4 (173)
	I do not know	27.3 (117)

**Table 3:** Classification of participants according to monkeypox knowledge level.

Knowledge Level	% (Number)
Adequate knowledge (score ≥ 5)	60.7 (260)
Inadequate knowledge (score < 5)	39.3 (168)

**Table 4:** Participants’ general attitudes to, opinions on, and awareness of the MP vaccine.

Parameters	Category	% (Number)
Q1: In order to control monkeypox infection, do you think the vaccine is necessary?	Yes	65.7 (281)
	No	13.1 (56)
	I do not know	21.3 (91)
Q2: Are you concerned about the possible side effects of monkeypox vaccines?	Yes	53.3 (228)
	No	22 (94)
	I do not know	24.8 (106)
Q3: Are you worried about the effectiveness of monkeypox vaccines?	Yes	48.4 (207)
	No	31.1 (133)
	I do not know	20.6 (88)
Q4: Are you going to encourage your friends and family to get vaccinated against monkeypox?	Yes	50.7 (217)
	No	18.5 (79)
	I do not know	30.8 (132)
Q5: Can you trust the safety of vaccines?	Yes	43.5 (186)
	No	22.9 (98)
	I do not know	33.6 (144)
Q6: Are the benefits of receiving the vaccines greater than the risks?	Yes	54.9 (235)
	No	9.3 (40)
	I do not know	35.7 (153)
Q7: Is the vaccine effective in protecting against monkeypox?	Yes	48.4 (207)
	No	9.8 (42)
	I do not know	41.8 (179)
Q8: Do you agree to receive the monkeypox vaccines?	Yes	52.3 (224)
	No	18.9 (81)
	I do not know	28.7 (123)
Did you receive monkeypox vaccines?	Yes	23.1 (99)
	No	51.9 (222)
	I do not know	25 (107)
Q9: Would it be possible to have a global pandemic caused by monkeypox?	Yes	44.9 (192)
	No	18.5 (79)
	I do not know	36.7 (157)

**Table 5:** Classification of participants according to attitude towards monkeypox vaccination.

Attitude Category	% (Number)
Positive attitude (score ≥ 5)	47.9 (205)
Negative attitude (score < 5)	52.1 (223)

**Table 6:** The association between adequate knowledge and sociodemographic variables.

Variable	*p*-Value
Gender	0.605
Age group	0.070
Education level	0.001
Job title	0.086
Years of experience	0.986

**Table 7:** Association between positive attitudes and sociodemographic variables.

Variable	*p*-Value
Gender	0.299
Age group	0.172
Education level	0.078
Job title	0.008
Years of experience	0.018

**Table 8:** Association between vaccination status and sociodemographic variables.

Variable	*p*-Value
Gender	0.382
Age group	0.002
Education level	0.142
Job title	0.056
Years of experience	0.051

## Data Availability

The original contributions presented in this study are included in the article. Further inquiries can be directed to the corresponding author.

## Abbreviations

The following abbreviations are used in this manuscript:

MP	Monkeypox
HCWs	Healthcare workers
US	United States
WHO	World Health Organization
